# The Evaluation of the Relationship Between Intercondylar and Intercanine Distances, Maxillary Central Incisor Width, and Various Facial Forms: A Comparative Study

**DOI:** 10.7759/cureus.43551

**Published:** 2023-08-15

**Authors:** Amal Jassim, Shanoj RP, Nandakumar K, Lakshmi Radhakrishnan, Juraise MC, Aysha Mohamed Ali KP

**Affiliations:** 1 Department of Prosthodontics, Muslim Educational Society (MES) Dental College, Perinthalmanna, IND

**Keywords:** maxillary central incisor width, intercondylar distance, intercanine distance, facial forms, face form indicator

## Abstract

Background

The intercondylar distance is a predictable and invariable parameter, which is not influenced by the soft tissue limitations and resorption unlike comparable anatomical markers. Limited studies are available on the use of intercondylar distance for the selection of teeth arrangement and its relationship with varying face forms.

Aim

The study aimed to evaluate the relationship between intercondylar distance and maxillary intercanine tip distances and central incisor width in square, tapering, and ovoid facial forms.

Materials and methods

The comparative cross-sectional study was performed between January 2021 and August 2022. A convenience sampling strategy was used to include subjects between the ages of 18 and 40 years who had all of their natural teeth. The facial forms of the subjects were detected using a face form indicator and grouped into ovoid (group A), tapering (group B), and square (group C) forms comprising 63 subjects in each group. The intercondylar and intercanine distances and maxillary central incisor width were measured using a digital caliper. The Statistical Package for Social Sciences (SPSS) software (IBM SPSS Statistics, Armonk, NY) was employed to determine the statistical difference between and across the groups using one-way analysis of variance (ANOVA) and post hoc analysis, respectively. The correlation between the variables was determined by the Pearson correlation test.

Results

The average age of the subjects was 24.55±3.47 years, and the age distribution was found to be statistically insignificant between the groups (p=0.63). The study consisted of 21% of males and 79% of females. The mean intercondylar distance was 125.2 mm in ovoid, 123.1 mm in tapering, and 125.9 mm in square face forms (p<0.01). The mean intercanine distance was 34.82 mm for ovoid, 37.11 mm for tapering, and 37.04 mm for square facial forms (p<0.01). Similarly, the mean central incisor width was 9, 7.84, and 8.51 mm for ovoid, tapering, and square facial forms, respectively (p<0.01). The ratio of intercondylar and intercanine distances in ovoid, tapering, and square faces was 1:3.59, 1:3.31, and 1:3.39, respectively. The ratio of intercondylar and central incisor width was 1:13.9, 1:15.7, and 1:14.7 for the groups A, B, and C, respectively. There was a statistically significant negative correlation between square and ovoid incisor width, square intercanine and ovoid incisor width, tapering incisor width and intercondylar distance, tapering intercanine and intercondylar distances, and tapering incisor width and square intercanine distance. The relationship between intercondylar and intercanine distances and the central incisor width was also revealed to be statistically highly significant (p<0.01).

Conclusion

When face form is taken into account, tapering face form shows more positive result for the relationship of intercondylar distance with intercanine distance and central incisor width. In patients with edentulous conditions, the intercondylar distance may offer useful measurements for tooth selection.

## Introduction

Over the years, dentists have suggested norms, standards, and guidelines for proper tooth selection and placement. However, selecting and arranging teeth with natural and aesthetically pleasing form and function for edentulous patients are elusive and challenging endeavors. No universally acceptable method has been established to meet this end [1]. The maxillary central incisor was the most critical tooth in the anterior teeth selection process because it is the most noticeable tooth from the frontal aspect and serves as the best assertion of patient age. The lateral incisor indicates the gender of the patient, and the canine displays the patient's dynamism [2]. Based on the theory of typal form, facial form is used as a reference standard for relating facial harmony with central incisors. The facial forms have been classified as square, tapering, and oval forms, and it came to light that the facial contours, when flipped over, may match the maxillary central incisor, producing aesthetically pleasing results [3,4]. However, a study conducted in a population of Zenica, Bosnia and Herzegovina, disclosed a controversial result and disapproved of the typal theory of relating face and tooth form [5].

Many approaches for selecting prosthetic teeth have been laid out in the scientific community. The dependability of commonly used anatomical markers such as intercommissural length, interpupillary distance, and interalar and bizygomatic dimensions has been a subject of interest for many years. These correlations were found to be successful when utilized together, although distinctions based on race and gender emerged when anatomical parameters were assessed separately. Anatomical landmarks that remain unaltered throughout life are the helping hands for anterior teeth selection. Intercondylar distance has been regarded as a stable landmark that is not hampered by resorption [6-8]. It has been documented that the ratio of intercondylar distance with maxillary intercanine distance was estimated to be 1:3.39, which is regarded as a reliable guideline for complete denture teeth arrangement. This ratio can be applied to the maxillary anterior teeth selection [9].

The intercondylar distance is a predictable and invariable parameter, which is not influenced by the soft tissue limitations and resorption unlike comparable anatomical markers. This method can be used in patients with nasal defects and eye defects where interpupillary and interalar width are not reliable [10]. Limited studies are available on the use of intercondylar distance for the selection of teeth arrangement and its relationship with varying face forms. The intercondylar distance may become useful when other parameters are not present or in conjunction with the other measurements. Thus, the study was aimed to evaluate the relationship between intercondylar distance and maxillary intercanine tip distances and central incisor width in square, tapering, and ovoid facial forms.

## Materials and methods

The comparative cross-sectional study was carried out between January 2021 and August 2022 after approval from the Institutional Ethics Committee of the Muslim Educational Society (MES) Dental College (IEC/MES/58/2020), complying with the standards of the Helsinki Declaration. A convenience sampling strategy was used to include subjects between the ages of 18 and 40 years who had all of their natural teeth after obtaining their informed consent. Individuals with any kind of facial asymmetry, malocclusion such as midline diastema, crowding/spacing, retained deciduous teeth, carious anterior teeth, prior history of temporomandibular dysfunction/orthodontic/prosthodontic treatment, and/or wasting diseases were excluded from the study. The mean and standard deviation (SD) of the intercondylar distance in tapered (µ1) and ovoid (µ2) facial forms were standardized as 0.31±0.02 and 0.32±0.02, respectively.

**Figure 1:**
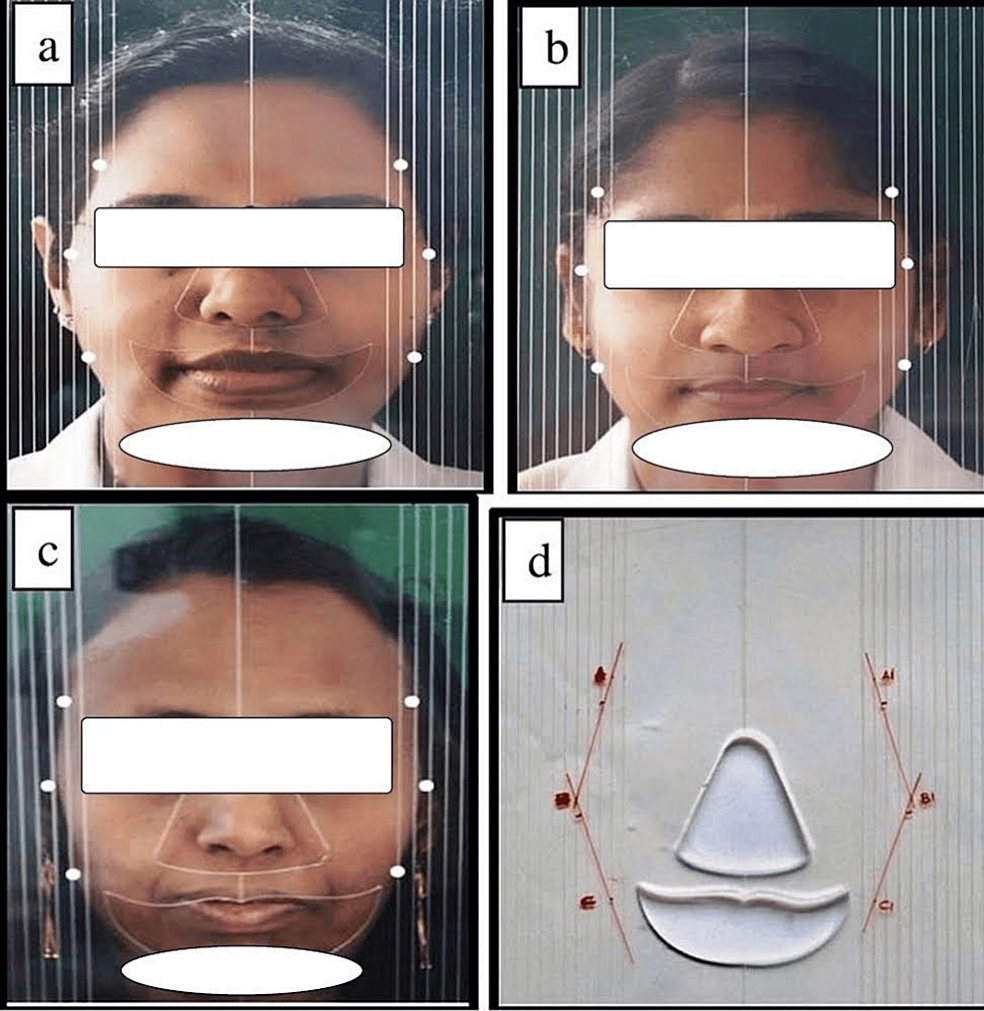
Grouping of various facial forms among the study subjects (a) Reference points on the facial form indicator for ovoid face form, (b) reference points marked on the indicator for square face form, (c) reference points marked on the indicator for tapering face form, and (d) lines joining reference points on transparent paper

The facial form of the subjects who met the inclusion criteria was detected clinically with the help of a customized facial form indicator, which is 30×30 cm, made of a flat 2 mm-thick plexiglass sheet. The reliefs for prominent facial structures were made. A straight line was drawn in the central region of the plate to define the sagittal plane. The plexiglass sheet was engraved with lines running parallel on the two sides separated by a 5 mm space. Each facial form was identified by juxtaposing these straight lines to the side of the individual's face (Figure 1).

The assessment of different facial forms, ovoid, square, and tapering, plays a crucial role in treatment planning. An ovoid facial form is characterized by balanced, soft contours. Square facial forms have proper angular features, harmonizing with a well-defined jawline and broad forehead. Tapering facial forms feature a narrower forehead and slender chin. The individual being examined was sitting upright, and the facial form indicator was positioned tightly to the face and perpendicular to the horizontal axis. The midline of the facial form indication was aligned to the facial midline and parallel to the coronal and horizontal planes of the face. Six points of reference (A, A1, B, B1, C, and C1) have been designated on the left and right sides of the facial form indicator to denote the greatest dimensions at the forehead, between the zygoma, and between the mandibular angles, which correspond to the upper, middle, and one-third of the face, respectively. After removing the facial form indicator, those reference marks were plotted on a translucent paper laid over the indicator. A line was drawn connecting reference points A, B, and C. Similarly, A1, B1, and C1 were connected to each other to generate the contour form of the face in each subject. Thus, the various facial forms of the study subjects were then grouped into three categories as groups A, B, and C comprising ovoid, tapering, and square facial forms, respectively.

The condyles were then identified utilizing the Beyron point, which is defined as a point 13 mm ahead to the posterior border of the tragus on a line stretching from the center of the tragus to the outer corner of the eye. A round-ended spreading caliper was used for measuring the intercondylar distance. The degree of convergence was defined as the angle established between the vertical line denoting the greatest width of the face and the middle, upper, or lower third of the face. Relying on the perception and the harmonious balance of proportion, the following standards were proposed aiming at the categorization of the face form [3]. The degree of convergence for square face was <5°, and that of the ovoid and tapering face was 5°-12° and greater than 13°.

After determining the face form, maxillary arch impression was made, and the intercanine tip distance between the two canine cusps and the maximum width from mesial and distal contact points of maxillary central incisor were determined with a digital caliper on the plaster cast. The intercondylar and intercanine distances and mesiodistal width of the maxillary central incisors were then compared. The average of three measurements were recorded.

Statistical analysis

The Statistical Package for Social Sciences (SPSS) version 24.0 (IBM SPSS Statistics, Armonk, NY) at p≤0.05 was utilized to calculate the mean±SD for the quantitative variables. The normality of the data was assessed using the Shapiro-Wilk test. The difference between and across the groups was computed using one-way analysis of variance (ANOVA) and post hoc analysis, respectively. The correlation between the variables was determined by the Pearson correlation test.

## Results

The study comprised 189 subjects categorized into groups A, B, and C with 63 subjects in each group based on the facial forms ovoid, tapering, and square, respectively. The age of the study sample was between 18 and 40 years with an average age of 24.55±3.47 years. The age distribution of the sample was found to be statistically insignificant between the groups (p=0.63). The study sample consisted of 21% (n=40) of males and 79% (n=149) of females. The average intercondylar and maxillary intercanine distances and width of the maxillary central incisors of the study sample were 124.6±0.28, 36.3±2.02, and 8.4±0.75 mm, respectively.

Table 1 illustrates the mean intercondylar distance, maxillary intercanine distance, and the maxillary central incisor width among the three facial forms of the sample.

**Table 1:** The mean intercondylar and intercanine distances and central incisor width among the three study groups HS, highly significant; NS, not significant; SD, standard deviation

Variables		Ovoid (A)	Tapering (B)	Square (C)	p-value	A versus B	A versus C	B versus C
Intercondylar distance	Mean±SD	12.52±0.11	12.31±0.13	12.59±0.41	0.001	HS	NS	HS
Intercanine distance	Mean±SD	34.82±1.76	37.11±1.43	37.04±1.94	0.001	HS	HS	NS
Central incisor width	Mean±SD	9±0.01	7.84±0.78	8.51±0.64	0.001	HS	HS	HS

The mean intercondylar distance was 125.2 mm in ovoid, 123.1 mm in tapering, and 125.9 mm in square face forms. The mean intercanine distance was 34.82, 37.11, and 37.04 mm for groups A, B, and C, respectively. Similarly, the mean central incisor width was 9, 7.84, and 8.51 mm for ovoid, tapering, and square facial forms, respectively. One-way ANOVA revealed a statistically highly significant difference between the groups A, B, and C with respect to intercondylar and intercanine distances and incisor width. In the context of intercondylar distance, a highly significant difference was observed between ovoid and tapering and tapering and square facial forms (p<0.01). Furthermore, a statistically highly significant difference was noticed between ovoid and tapering and ovoid and square facial forms with respect to intercanine distance (p<0.01). Further, the width of the central incisors showed a statistically highly significant difference between ovoid and tapering, ovoid and square, and tapering and square face forms.

Table 2 shows the ratio between intercondylar and intercanine distances and intercondylar and central incisor width.

**Table 2:** Ratio between intercondylar and intercanine distances and intercondylar and central incisor width

Facial form	Intercondylar and intercanine distances	Intercondylar and central incisor width
Ovoid	1:3.59	1:13.9
Tapering	1:3.31	1:15.7
Square	1:3.39	1:14.7

The ratio of intercondylar and intercanine distances in ovoid, tapering, and square faces was 1:3.59, 1:3.31, and 1:3.39, respectively. On the other hand, the ratio of intercondylar and central incisor width was determined as 1:13.9, 1:15.7, and 1:14.7, respectively, for ovoid, tapering, and square facial forms. This ratio can be applied for the teeth selection process among patients of different face forms.

Table 3 depicts a statistically significant negative correlation between square and ovoid incisor width and square intercanine and ovoid incisor width (r=-0.30; p=0.02).

**Table 3:** Correlation between intercondylar and intercanine distances and central incisor width among the three study groups with ovoid, tapering, and square facial forms

Variables		Ovoid condylar	Ovoid canine	Ovoid incisor	Tapering condylar	Tapering canine	Tapering incisor	Square condylar	Square canine	Square incisor
Ovoid condylar	r	1	-0.12	-0.02	0.07	-0.15	-0.11	-0.19	-0.23	-0.04
	p-value		0.36	0.91	0.58	0.26	0.40	0.14	0.07	0.74
Ovoid canine	r	-0.12	1	0.01	-0.17	0.19	0.05	0.13	0.2	0.006
	p-value	0.36		0.92	0.18	0.14	0.72	0.31	0.12	0.96
Ovoid incisor	r	-0.02	0.01	1	0.19	-0.19	0.02	-0.001	-0.07	-0.30
	p-value	0.91	0.92		0.15	0.15	0.86	0.99	0.60	0.02
Tapering condylar	r	0.07	-0.17	0.19	1	-0.58	-0.36	-0.09	0.16	0.05
	p-value	0.58	0.18	0.15		0.001	0.004	0.48	0.20	0.68
Tapering canine	r	-0.15	0.19	-0.19	-0.58	1	-0.01	0.04	0.005	-0.03
	p-value	0.26	0.14	0.15	0.001		0.94	0.76	0.97	0.83
Tapering incisor	r	-0.11	0.05	0.02	-0.36	-0.01	1	0.01	-0.3	0.03
	p-value	0.40	0.72	0.86	0.004	0.94		0.91	0.02	0.82
Square condylar	r	-0.19	0.13	-0.001	-0.09	0.04	0.01	1	-0.09	0.17
	p-value	0.14	0.31	0.99	0.48	0.76	0.91		0.48	0.18
Square canine	r	-0.23	0.20	-0.07	0.16	0.005	-0.30	-0.09	1	-0.15
	p-value	0.07	0.12	0.60	0.20	0.97	0.02	0.48		0.23
Square incisor	r	-0.04	0.01	-0.30	0.05	-0.03	0.03	0.17	-0.15	1
	p-value	0.74	0.96	0.02	0.68	0.83	0.82	0.18	0.23	

Also, negative correlation was observed between tapering incisor width and intercondylar distance (r=-0.36; p=0.004), tapering intercanine and intercondylar distance (r=-0.58; p=0.001), and tapering incisor width and square intercanine distance (r=-0.3; p=0.02).

Table 4 reveals the correlation between intercondylar and intercanine distances and central incisor width.

**Table 4:** Correlation between intercondylar and intercanine distances and central incisor width of the study sample

Variables		Intercondylar distance	Intercanine distance	Central incisor width
Intercondylar distance	r	1	-0.193	0.226
	p-value		0.008	0.002
Intercanine distance	r	-0.193	1	-0.330
	p-value	0.008		0.001
Central incisor width	r	0.226	-0.330	1
	p-value	0.002	0.001	

The Pearson correlation indicates a statistically highly significant negative correlation between intercondylar and intercanine distances (r=-0.19; p=0.01), positive correlation between intercondylar distance and central incisor width (r=0.23; p=0.002), and negative correlation between intercanine distance and incisor width (r=-0.33; p=0.01).

## Discussion

The guiding factors for the selection of artificial teeth include aesthetics, particularly for the replacement of the anterior teeth; efficient mastication; and pronunciation. The placement of prosthetic teeth in a full denture is governed by their relationship to facial anatomical features. It is suggested that these teeth be placed in the corresponding jaws in a manner similar as possible to their natural antecedents in order to restore function with artificial counterparts that mimic both in dimension and in position the missing natural teeth. However, the lack of pre-extraction documents is a major impediment to using this full denture construction procedure. Several consistent anatomical landmarks and their associated relationships are employed for the same goal in such cases [11,12].

Relying on a single anatomical landmark alone may result in errors. Many authors suggest combining various landmarks for teeth selection. Commonly used landmarks are interalar distance, incisive papilla, bizygomatic distance, ear dimensions, and intercanthal distances [13-15]. Very limited studies are available on the reliability of intercondylar distance as an anatomical landmark for anterior teeth selection [16]. The intercondylar distance is a predictable and invariable parameter and is not influenced by the limitations of soft tissues and resorption unlike comparable anatomical markers. This method can be used in patients with maxillofacial defects where other anthropometric measures are not reliable.

Studies on intercondylar and intercanine distances were done by Keshvad et al. [9], Debnath et al. [1], Lazić et al. [17], Shaikh et al. [10], Qamar et al. [18], and Shrestha et al. [19]. Keshvad et al. reported that the ratio of intercanine distance to intercondylar distance was 1:3.39. They determined the intercondylar distance by using a kinematic facebow and concluded that intercondylar distance was a reliable landmark for anterior teeth selection [9]. Kassab evaluated the relationships between maxillary intercanine distance, central incisor width, and facial parameters including interzygomatic, inner/outer canthus and interpupillary distances and also appraised the correlation among various types of facial forms such as leptoprosopic, euryprosopic, and mesoprosopic faces for the optimal selection of the anterior maxillary teeth. He found that leptoprosopic face had a higher correlation with anterior teeth measurements [2].

In the present study, the face forms were selected with the help of a customized face form indicator similar to that of Trubyte indicator, which was first made by Ashok and Ganapathy [3]. The clinical and photographic approaches disclosed insignificant differences between the categorization of face forms [3]. Henceforth, the present study was done using the customized facial form indicator, and the digital caliper was utilized to determine the intercanine distance and the central incisor width on a stone cast, as used by Varjao and Nogueira [20], Smith [21], and Keshvad et al. [9] in their studies. The mesiodistal diameter of the maxillary central incisors is crucial for anterior teeth selection since they are the most determining teeth in the arch with respect to aesthetics. Shillingburg et al. established that the combined mesiodistal diameter of the maxillary central incisors comprised 37% of the circumferential arch dimension between the distal surfaces of the maxillary canines. The cumulated width of the lateral incisors was 31%, and that of the canines was 32% of the distance [22].

Parajuli et al. [15] conducted a similar study to evaluate the relationship of intercondylar distance with maxillary intercanine distance and maxillary intermolar distance and concluded that intercondylar distance has a positive correlation with the maxillary intercanine and intermolar distances. Kurien et al. measured the distal aspect of each maxillary canine, across the labial surfaces of the anterior teeth, using brass wire and a caliper. Interalar and intercommissural width were measured with a vernier caliper, and it was concluded that interalar width revealed a significant relationship with circumferential arc dimension. Thus, the nasal dimension was more effective in determining the width of maxillary anterior teeth [23]. The mean intercondylar distance in the present study correlates with the findings of Lazić et al. [17]. However, the study findings by Keshvad et al. [9] and Debnath et al. [1] were lesser than that of the present study. The intercondylar distance found for square face was the highest, and the least was for tapering face form. Similarly, the mean intercanine distance in this study correlates with the findings of Debnath et al. [1] but is greater than that reported by Keshvad et al. [9]. The mean value of intercanine distance was found to be higher for tapering and least for ovoid face form.

The maxillary central incisor is an ideal reference point for the replacement of lost tooth. Studies by Dwivedi et al. [24], Vasanth Kumar et al. [25], Sellen et al. [26], Pound [27], Shetty et al. [28], and Pisulkar et al. [29] have used maxillary central incisor as a reference point for anterior teeth selection. In the present study, the relationship of maxillary central incisor width with intercondylar distance was also evaluated and was found significant. It was also found that mean central incisor width was higher for ovoid face form and lowest for tapering face form. Keshvad et al. [9] formulated a ratio for selecting anterior teeth using intercondylar distance. Similarly, in the present study, the ratio was calculated between the intercondylar and intercanine distances and intercondylar distance and central incisor width, which was in agreement with the ratio obtained from the previous study for intercondylar distance and intercanine distance.

In the current study, the intercondylar distance was found to be more significantly correlated with tapering face form. This indicates that in tapering face form, the intercondylar distance can be used to select the anterior teeth in edentulous patients. This was concurrent with the study conducted by Ibrahimagić et al. [5] who documented that face form alone cannot be relied for the selection of anterior teeth and different face form has variations in values of intercondylar distance and anterior teeth width. Keshvad et al. [9] conducted the study without grouping on the basis of face forms and found that intercondylar distance can be employed for anterior teeth selection. But the current study conducted by categorizing the sample based on the face forms found that intercondylar distance is more related to anterior teeth selection in tapering face form. Certain similar studies reported an insignificant difference between the intercondylar width determined using an arbitrary and a kinematic facebow [1,9,10,17-19]. They further documented that arbitrary facebow could effectively record the intercondylar distance. In our study, the intercondylar distance was measured using a round-ended spreading caliper, which was placed by locating the condyles using the Beyron point.

The findings of the current study suggest the significant relation between the stable intercondylar distance and intercanine distance and maxillary central incisor width, which serves as a more practical means for prosthetic teeth selection. The approximate intercanine distance could be calculated by dividing the distance between two condyles with 3.59 for ovoid, 3.31 for tapering, and 3.39 for square face forms [9,10]. Also, this method of selecting anterior teeth is more accurate for tapering face forms. It is suggested that the intercondylar and interdental widths serve as an additional aid for the rehabilitation of the missing tooth especially without pre-extraction records [30].

The skeletal and ethnic variations were not addressed to generalize the study findings. Also, the resiliency of the soft tissues, method of impression making, and plaster cast can cause alterations in the external tissue measurements. Furthermore, this study was conducted within an institutional setting, and only patients aged 18-40 years were considered. As a result, the findings may only apply to a restricted group in the specified age range. The study will be more comprehensive and needs to be corroborated by integrating a broad population size dispersed across the entire nation. This would aid in the generation of a combination of factors across different anthropological metrics for utilization among the subpopulation of the country. 

The limitations of the study include the following: the study's sample size might be considered relatively small for drawing generalized conclusions, and the demographics of the subjects (age range, gender distribution, ethnic backgrounds, etc.) were not thoroughly discussed. This could affect the external validity of the study's findings. The study used a convenience sampling strategy, which might introduce selection bias and limit the generalizability of the results to a broader population. The method used to categorize subjects into facial forms using a face form indicator might be subjective and not universally accepted. The accuracy and consistency of measurements taken using digital calipers could be influenced by the skills of the person conducting the measurements.

## Conclusions

The intercondylar and intercanine distances and central incisor widths are showing a positive correlation and are significant when face form is not considered. When face form is taken into account, tapering face form shows a more positive result for the relationship of intercondylar distance with intercanine distance and central incisor width. The intercondylar and maxillary intercanine ratio could be used for maxillary anterior teeth selection for tapering and square face forms. The intercondylar dimension could be an effective parameter for teeth selection in edentulous individuals.
